# Construction and evaluation of a model to identify early postoperative kinesiophobia in patients with lumbar degenerative diseases: a cross-sectional study

**DOI:** 10.3389/fresc.2026.1752970

**Published:** 2026-04-08

**Authors:** Zhumei Fang, Honghui Zhang, Ziping Zhang, Jianqing Zheng, Huifen Zhao

**Affiliations:** 1 Department of Orthopedics, The Second Affiliated Hospital of Fujian Medical University, Quanzhou, China; 2 Fujian Provincial Health Commission, Fuzhou, China; 3 Department of Radiation Oncology, The Second Affiliated Hospital of Fujian Medical University, Quanzhou, China; 4 Department of Nursing, The Second Affiliated Hospital of Fujian Medical University, Quanzhou, China

**Keywords:** kinesiophobia, lumbar degenerative diseases, nomogram, prediction model, web-based calculator

## Abstract

**Objectives:**

This study investigated the prevalence of early postoperative kinesiophobia in patients with lumbar degenerative diseases (LDDs) who had undergone surgery. The aim was to identify associated factors influencing kinesiophobia, and to develop a nomogram to provide a screening tool to identify early postoperative kinesiophobia in patients with LDDs.

**Methods:**

A cross-sectional study was designed to investigate the prevalence of kinesiophobia in patients with LDDs. Data on 301 patients with LDDs, who had undergone lumbar fusion surgery in the Second Affiliated Hospital of Fujian Medical University from January 2023 to August 2023, was used as the training set, while data on 101 patients at the Quanzhou Orthopedic-Traumatological Hospital from September 2023 to November 2023 was used as the test set. A model was constructed by using the results of univariate logistic regression analyses, and was assessed in terms of the accuracy, recall, kappa value, and F1 score. The performance evaluation of this model is based on its ability to distinguish and calibrate early postoperative kinesiophobia, as well as its clinical practicality. Moreover, the entire process was implemented according to the Transparent Reporting of a multivariable prediction model for Individual Prognosis or Diagnosis checklist.

**Results:**

The prevalence of kinesiophobia among patients with LDDs in the entire dataset was 62.69% (252/402), with a prevalence of 61.13% (184/301) and 67.33% (68/101) in the training and test sets, respectively. Age, sex, intensity of pain, anxiety, depression, self-efficacy, and social support were identified as independent associated factors in the multivariate logistic regression analysis, and a nomogram was developed based on them. The area under the curve, accuracy, sensitivity, and specificity of the model on the training and test sets were 0.931 (0.890, 0.972) and 0.874 (0.781, 0.966), 0.937 (0.904, 0.959) and 0.861 (0.781, 0.916), 0.957 (0.917, 0.978) and 0.838 (0.733, 0.907), and 0.906 (0.839, 0.947) and 0.909 (0.764, 0.969), respectively. Furthermore, the values of the Hosmer-Lemeshow test on the training and test sets were *χ*^2^ = 8.32, *df* = 13, and *P* = 0.822, and *χ*^2^ = 11.63, *df* = 13, and *P* = 0.235, respectively.

**Conclusion:**

The proposed model accurately identified early postoperative kinesiophobia in patients with LDDs based on their age, sex, intensity of pain, anxiety, depression, self-efficacy and social support. The model delivered sound performance in terms of discrimination, calibration, and clinical applicability. Furthermore, the nomogram model and online web-based calculator were easy to use for nurses, and enabled them to quickly identify early postoperative kinesiophobia in patients with LDDs.

## 1 Introduction

Lumbar degenerative diseases (LDDs), also called lumbar degenerative disk diseases or degenerative lumbar spine diseases, include lumbar disk herniation, lumbar spondylolisthesis, and lumbar spinal stenosis (1, 2). LDDs are accompanied by a variety of clinical symptoms, including lumbago and leg pain of varying severity, weakness of the lower limbs, lower back pain, and claudication, that can lead to a decline in the quality of life (3). The prevalence of LDDs is increasing and 266 million patients are annually diagnosed with them worldwide (2). The main methods used to treat LDDs include conservative and surgical treatments (4). In general, surgical treatment is recommended when conservative treatment is ineffective for a month or if the disease is still progressing (5). The success of surgical treatment for patients with LDDs is 60%–90%, but the prognoses of such patients is unsatisfactory, as up to 40% of those who have undergone surgery continue to suffer from persistent pain, poor lumbar spine function, and a low quality of life (6). Strong postoperative pain can easily cause patients to become hesitant to engage in daily rehabilitation exercises, for fear that the resulting pain will cause secondary injuries (7, 8).

Kinesiophobia refers to an excessive and irrational fear of physical activity or movement, and is common in patients who have suffered a painful physical injury that leads to increased sensitivity to their own pain (9). About 45%–60% of patients with LDDs suffer from kinesiophobia after surgery, and it is the main cause of disability in patients with acute and chronic back pain (10). As the degree of kinesiophobia rises, the extent to which patients with LDDs comply with their rehabilitation training decreases. Insufficient postoperative rehabilitation training results in the disuse-induced atrophy of the lumbar muscles, increased risk of nerve root adhesions, and deep vein thrombosis that can affect the rehabilitation of patients with LDDs (11, 12). Research has also shown that kinesiophobia can act as an independent factor that directly contributes to postoperative lumbar spine incapacitation, functional decompensation, and longer-lasting pain such that it has an impact on the outcomes of rehabilitation (13). Early intervention in case of kinesiophobia can promote pain relief and functional rehabilitation in patients with LDDs (14). The early identification of patients at a high risk of kinesiophobia and appropriate intervention can better alleviate postoperative pain and improve the postoperative prognosis of patients with LDDs.

Prevalent research on kinesiophobia is limited in terms of accurately predicting its risk. To the best of the authors' knowledge, no tool is currently available for identifying early postoperative kinesiophobia in patients with LDDs. Therefore, this study aimed to screen factors influencing kinesiophobia after surgery in patients with LDDs, establish a model to identify early postoperative kinesiophobia, and subject it to internal and external validation. The aim was to provide clinical nurses with an accurate tool for the early identification of postoperative kinesiophobia, and to provide theoretical guidance for preventative research in the field.

## 2 Methods

### 2.1 Study design and participants

A cross-sectional study was designed to investigate the prevalence of kinesiophobia in patients with LDDs. A continuous sampling plan was used to ensure the reliability of the results. Patients in the Department of Orthopedics of the Second Affiliated Hospital of Fujian Medical University who had undergone lumbar fusion surgery (LFS) from January 2023 to August 2023, and those in the Department of Orthopedics of Quanzhou Orthopedic–Traumatological Hospital who had been subjected to the same procedure from September 2023 to November 2023, were enrolled in the study. All subjects met the following criteria: (1) age ≥18 years; (2) the patients were undergoing LFS for back pain and/or leg pain owing to degenerative diseases; (3) the degenerative diseases included lumbar disk herniation, lumbar spinal stenosis, and lumbar spondylolisthesis, had been diagnosed and confirmed through imaging examinations, and the patients had been in pain for more than 3 months; and (4) the patients had reading comprehension and the capability of speech. The criteria for exclusion were as follows: (1) patients who had serious chronic diseases (such as severe liver disease, kidney disease, heart disease, and rheumatic disease), (2) patients with lumbar spine trauma, spinal tumors, infections, pseudo-arthritis, physical disabilities, and cauda equina syndrome, (3) patients with mental illness and/or hearing impairment, and (4) patients who were comatose, or had been transferred to the intensive care unit after surgery. A first survey of the patients was conducted 3 days after they had undergone LFS for the first time.

**Figure 1:**
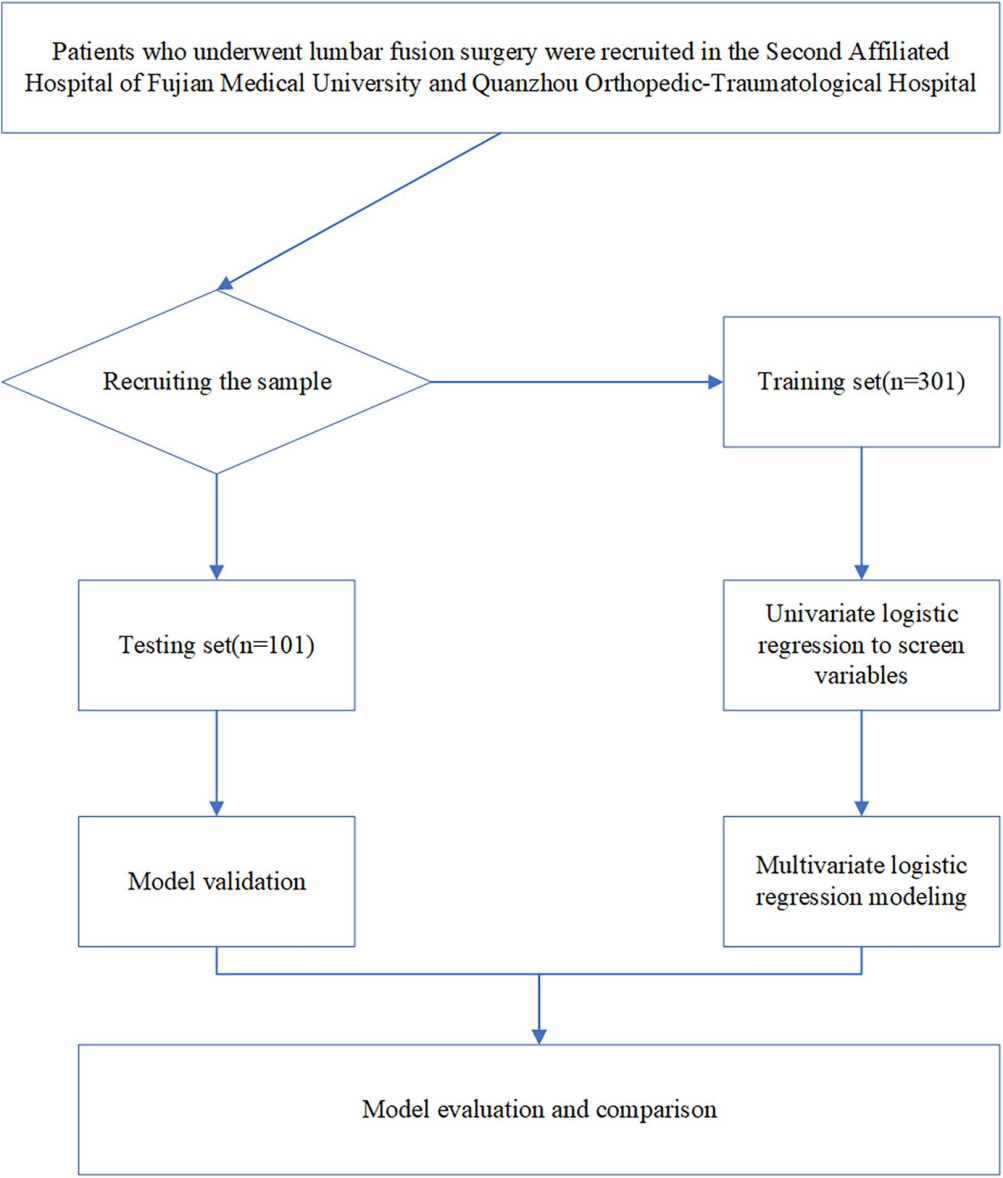
Flowchart of the design of this study.

The event per variable (EPV) was used to estimate the sample size in logistic regression analysis (15). A total of 16 preset independent variables were thus identified. The prevalence of postoperative kinesiophobia was 60% (24/40) according to the preliminary survey. Considering the failure rate of the survey of 10%, the size of the sample required to formulate a logistic regression model for the study would have featured at least (10 × 16 ÷ 0.6) ÷ (1 − 0.1) = 296 cases. Finally, a total of 401 cases were considered. Among them, 301 patients were enrolled from Department of Orthopedics of the Second Affiliated Hospital of Fujian Medical University, and were put into the training set. 101 patients were enrolled from Department of Orthopedics of Quanzhou Orthopedic–Traumatological Hospital, and were put into the test set. A flowchart of the design of this study is shown in Figure 1.

### 2.2 Data collection

Two investigators who had undergone the same training surveyed the recruited patients in person by using a paper questionnaire on the third day after their surgery. Prior to the formal survey, a pilot study was conducted with the same questionnaire to assess whether its questions or items were easy to understand, and if there was any technical problem in 40 cases of patients with LDDs. Before conducting the survey, the investigators explained to the patients the purpose of the study, its content, and the time required to fill out the questionnaire. They also assured the subjects that their data would be anonymized. The questionnaires were then distributed to the eligible patients. The investigators were responsible for answering any questions that the subjects had. To make sure that the questionnaire was administered under comparable postoperative analgesic statuses and restrictions on mobility, all surveys were conducted between 8:00 and 10:00 a.m. on the morning of the third day after surgery. Once the patients had completed the questionnaires, the investigators checked them on the spot for completeness. A satisfactorily filled questionnaire contained no missing data, and thus no missing data were determined for any predictor or outcome in this study.

### 2.3 Study instruments

#### 2.3.1 Demographic characteristics and medical history

Potential predictor variables were initially identified and selected based on the relevant literature, and an assessment questionnaire was accordingly designed. The aim of the questionnaire was to collect general information on the subjects. It sought the following information: (1) socio-demographic characteristics, including sex, age, status of employment, body mass index (BMI), level of education, marital status, place of residence, per capita monthly household income, and reimbursement for medical expenses; and (2) medical history, including disease history, and the site, duration, and severity of pain.

#### 2.3.2 Visual analog scale (VAS)

Postoperative pain was measured by using the self-reported visual analog scale (VAS) (16), which was scored in a range from 0 (no pain) to 10 points (maximal intensity of pain). Higher scores reflected more severe pain, and a score of higher than four points was considered to be indicative of moderate to severe pain.

#### 2.3.3 General self-efficacy scale (GSES)

This scale was developed by Schwarzer et al. in 1981 (17). The initial version contained 20 items but was subsequently reduced to 10 items. The GSES is a four-point Likert scale with a total score of 40. The higher the score on the GSES, the higher the level of self-efficacy of the relevant subject. The Cronbach's alpha coefficient of the GSES was 0.870.

#### 2.3.4 Tampa scale for kinesiophobia (TSK)

The degree of kinesiophobia was measured by using the TSK, which was developed by Swinkels-Meewisse et al. (18). The TSK contained 17 items, and was a four-point Likert scale (1 = strongly disagree, 4 = strongly agree) with a total score ranging from 17 to 68. A score greater than or equal to 37 indicated kinesiophobia, while higher scores reflected greater fear of movement (19, 20). The TSK was translated into Chinese by Hu and used to survey patients with degenerative low back pain. It is as an important tool for assessing kinesiophobia (21). The Chinese version of the TSK has demonstrated a high reliability, with a value of Cronbach's alpha of 0.778 and a reliability of re-testing of 0.960 (21).

#### 2.3.5 Social support rating scale (SSRS)

The SSRS was used to assess the degree of social support for the subjects. It consists of 10 items over three dimensions: subjective support (four items), objective support (three items), and utilization of social support (three items) (22). The higher the score on the SSRS is, the stronger was the social support system available to the corresponding subject. The SSRS had acceptable psychometric properties (Cronbach's alpha = 0.82) for measuring social support (23).

#### 2.3.6 Hospital anxiety and depression scale (HADS)

This scale was developed by Zigmond et al. (24). It consisted of 14 items with two subscales for anxiety and depression, of which seven items (1, 3, 5, 7, 9, 11, and 13) were used to rate anxiety while the remainder (2, 4, 6, 8, 10, 12, and 14) was used to rate depression. It was a four-point Likert scale (0–3) with a total score of 0–21 points. Higher scores represented higher levels of anxiety or depression in individuals. If both subscales had scores higher than nine points, the relevant subject was assumed to have both an anxious and a depressed state. A normal psychological state was scored from 0 to 7 points, mild anxiety and depression yield scores of 8–10 points, moderate anxiety and depression were represented by a score of 11–14 points, while severe anxiety and depression were represented by the range of 15–21 points. The Cronbach's alpha for this scale was 0.879.

### 2.4 Statistical analysis

Statistical analyses were performed by using R software (version 4.3.1). Quantitative data that conformed to a normal distribution were expressed in terms of $\bar{x}\pm s$, and intergroup comparisons were conducted by using independent sample *t*-tests. Count data were expressed as percentages, while intragroup comparisons were conducted by using the chi-squared test or Fisher's exact probability test. Univariate logistic regression analysis was used to determine the potential factors associated with kinesiophobia. All factors with *Р* < 0.05 in the univariate analysis were included in further multifactorial logistic regression analyses. The screening model was developed by using multifactorial logistic regression. The score for each variable was calculated according to multivariate logistic regression, and the predictive probability of kinesiophobia was determined by summing the scores for each variable. The software packages “rms” and “dynnom” were used to draw a nomogram based on the results of multivariate logistic regression. ROC curves were used to assess the efficiency of identification of the model, calibration was used to measure how well the probabilities of screening matched the actual results, while the Hosmer–Lemeshow test was used to evaluate the capability for calibration, with *Р* > 0.05 representing satisfactory calibration. The clinical utility of the screening model was assessed based on net benefit at different threshold probabilities by using Decision Curve Analysis (DCA) and clinical impact curves. The optimal cut-off value was determined based on the analysis of the ROC curves of the training cohort when the Youden index (sensitivity + specificity − 1) reached its maximum value. The accuracy of screening of the model was assessed through differentiation and calibration, and a corresponding receiver–operating characteristic (ROC) curve was plotted. The area under the curve (AUC) was also calculated to assess differentiation, and ranged from 0% to 100%. Higher values reflected better capability of differentiation by the model. Internal validation was performed by using bootstrap resampling (1,000 replications) to estimate the optimism-corrected AUC and the calibration.

### 2.5 Ethical considerations

All patients participating in this study provided their signed informed consent, and were allowed to withdraw from it at any time. All data were treated as confidential. This study was approved by the Ethics Committee of the Second Affiliated Hospital of Fujian Medical University (2022-342).

## 3 Results

### 3.1 Characteristics of participants

A total of 420 questionnaires were distributed, of which 310 were provided to subjects in the Second Affiliated Hospital of Fujian Medical University (the training set) and 110 to those in the Quanzhou Orthopedic-Traumatological Hospital (test set). A total of 402 valid questionnaires were recovered, of which 301 questionnaires were for the training set and 101 for the test set, with an effective rate of 95.71%. The rate of prevalence of kinesiophobia among respondents with LDDs was 62.69% in the entire set (252/402), with rates of 61.13% (184/301) and 67.33% (68/101) in the training and test sets, respectively. Data on the patients considered in the training and test sets are presented in Table 1. The distributions of samples in the training and test sets were consistent and comparable (*P* > 0.05). The panoramic data of the patients included in the study were presented in Supplementary Table S1.

**Table 1:** Panoramic data on patients considered in the training and test sets.

Variables	Variable levels	Total set (*n* = 402)	The test set (*n* = 101)	The training set (*n* = 301)	*t* value/*F* value	*P* value
TSK		41.65 ± 14.04	41.42 ± 14.05	41.73 ± 14.06	0.195	0.846
HADS_Anxiety		10.36 ± 4.93	9.98 ± 5.01	10.48 ± 4.90	0.875	0.383
HADS_Depression		10.44 ± 5.17	10.65 ± 5.35	10.37 ± 5.12	0.473	0.637
GSES		22.58 ± 6.75	22.69 ± 6.98	22.54 ± 6.68	0.187	0.852
SSRS		36.88 ± 12.66	36.82 ± 12.75	36.90 ± 12.65	0.054	0.957
Age		60.35 ± 14.85	62.01 ± 15.13	59.79 ± 14.74	1.286	0.200
BMI		23.98 ± 2.29	24.14 ± 2.19	23.92 ± 2.33	0.853	0.395
VAS		6.09 ± 1.87	5.88 ± 1.89	6.17 ± 1.86	1.317	0.189
Height		1.72 ± 0.10	1.73 ± 0.10	1.72 ± 0.10	0.485	0.628
Weight		62.63 ± 7.47	63.00 ± 7.63	62.51 ± 7.43	0.560	0.576
Kinesiophobia	No	150 (37.31)	33 (32.67)	117 (38.87)	1.242	0.265
	Yes	252 (62.69)	68 (67.33)	184 (61.13)		
Sex	Female	143 (35.57)	40 (39.60)	103 (34.22)	0.957	0.328
	Male	259 (64.43)	61 (60.40)	198 (65.78)		
Years Of Pain	<3 years	141 (35.07)	33 (32.67)	108 (35.88)	0.962	0.618
	3 ± 5 years	175 (43.53)	43 (42.57)	132 (43.85)		
	>5 years	86 (21.39)	25 (24.75)	61 (20.27)		
Work Status	Employed	181 (45.02)	46 (45.54)	135 (44.85)	1.418	0.492
	Unemployed	79 (19.65)	16 (15.84)	63 (20.93)		
	Retirement	142 (35.32)	39 (38.61)	103 (34.22)		
Education	Elementary education	183 (45.52)	48 (47.52)	135 (44.85)	0.356	0.949
	Middle school	77 (19.15)	18 (17.82)	59 (19.60)		
	High school	60 (14.93)	14 (13.86)	46 (15.28)		
	Tertiary education	82 (20.40)	21 (20.79)	61 (20.27)		
Marriage	Unmarried	67 (16.67)	18 (17.82)	49 (16.28)	1.430	0.699
	Married	244 (60.70)	60 (59.41)	184 (61.13)		
	Widowed	67 (16.67)	19 (18.81)	48 (15.95)		
	Divorced or separated	24 (5.97)	4 (3.96)	20 (6.64)		
Residence	Rural	171 (42.54)	40 (39.60)	131 (43.52)	1.183	0.554
	Township	82 (20.40)	19 (18.81)	63 (20.93)		
	City	149 (37.06)	42 (41.58)	107 (35.55)		
Income	<1,000 Yuan	65 (16.17)	16 (15.84)	49 (16.28)	1.810	0.613
	1,000 ± 2,999 Yuan	171 (42.54)	43 (42.57)	128 (42.52)		
	3,000 ± 4,999 Yuan	82 (20.40)	17 (16.83)	65 (21.59)		
	>5,000 Yuan	84 (20.90)	25 (24.75)	59 (19.60)		
Medical Insurance	Fully self-funded	69 (17.16)	21 (20.79)	48 (15.95)	1.659	0.436
	Partially medical insurance	294 (73.13)	69 (68.32)	225 (74.75)		
	Fully medical insurance	39 (9.70)	11 (10.89)	28 (9.30)		
Procedure Type	Posterolateral fusion	94 (23.38)	24 (23.76)	70 (23.26)	2.639	0.451
	Posterior lumber interbody fusion	86 (21.39)	16 (15.84)	70 (23.26)		
	Transforaminal lumbar interbody fusion	115 (28.61)	31 (30.69)	84 (27.91)		
	Anterior lumbar interbody fusion	107 (26.62)	30 (29.70)	77 (25.58)		
Decompression	Yes	308 (76.62)	81 (80.20)	227 (75.42)	0.966	0.326
	No	94 (23.38)	20 (19.80)	74 (24.58)		

TSK, Tampa Scale for Linesiophobia; HADS_Anxiety, Hospital Anxiety and Depression Scale-Anxiety; HADS_Depression, Hospital Anxiety and Depression Scale-Depression; GSES, General Self-Efficacy Scale; SSRS, Social Support Rating Scale; BMI, Body Mass Index; VAS, Visual Analog Scale.

### 3.2 Univariate analyses of potential predictive factors of kinesiophobia

Univariate logistic regression analysis was performed on the training set to explore the independent factors associated with postoperative kinesiophobia, and the results are presented in Table 2. They showed that anxiety, depression, GSES, SSRS, age, BMI, VAS, sex, years of pain, employment status, education, and diagnosis for admission to the hospital all had a significant effect on kinesiophobia status (*P* < 0.05).

**Table 2:** Univariate analyses of the potential predictive factors associated with kinesiophobia in patients with LDDs.

Variables	Variable levels	Beta	SE of Beta	OR	Statistic	*P*
HADS_Anxiety		0.16	0.03	1.17 (1.11, 1.24)	5.668	<0.001
HADS_Depression		0.20	0.03	1.22 (1.15, 1.29)	6.854	<0.001
GSES		−0.18	0.02	0.83 (0.80, 0.87)	7.534	<0.001
SSRS		−0.17	0.02	0.84 (0.81, 0.87)	9.178	<0.001
Age		0.14	0.02	1.15 (1.11, 1.18)	8.735	<0.001
BMI		0.16	0.05	1.17 (1.06, 1.30)	3.054	0.002
VAS		0.42	0.07	1.52 (1.32, 1.76)	5.661	<0.001
Height		0.40	1.17	1.49 (0.15, 14.68)	0.344	0.731
Weight		0.00	0.02	1.00 (0.97, 1.04)	0.269	0.788
Sex	Female					
	Male	0.80	0.25	2.22 (1.36, 3.61)	3.203	0.001
Years of pain	<3 years					
	3–5 years	0.87	0.27	2.39 (1.41, 4.05)	3.222	0.001
	>5 years	0.61	0.33	1.84 (0.97, 3.50)	1.854	0.064
Work status	Employed					
	Unemployed	0.69	0.31	2.00 (1.09, 3.66)	2.234	0.025
	Retirement	3.25	0.46	25.80 (10.55, 63.12)	7.123	<0.001
Education	Elementary education					
	Middle school	−1.93	0.35	0.15 (0.07, 0.29)	5.456	<0.001
	High school	−2.23	0.39	0.11 (0.05, 0.23)	5.753	<0.001
	Tertiary education	−1.92	0.35	0.15 (0.07, 0.29)	5.484	<0.001
Marriage	Unmarried					
	Married	0.19	0.32	1.21 (0.64, 2.29)	0.591	0.554
	Widowed	0.68	0.43	1.98 (0.85, 4.58)	1.594	0.111
	Divorced or separated	0.41	0.55	1.51 (0.52, 4.45)	0.753	0.451
Residence	Rural					
	Township	0.46	0.32	1.59 (0.85, 2.97)	1.448	0.148
	City	0.37	0.27	1.44 (0.85, 2.44)	1.368	0.171
Income	<1,000 Yuan					
	1,000–2,999 Yuan	0.36	0.34	1.43 (0.73, 2.77)	1.047	0.295
	3,000–4,999 Yuan	0.48	0.39	1.62 (0.76, 3.44)	1.241	0.214
	>5,000 Yuan	0.40	0.39	1.49 (0.69, 3.21)	1.011	0.312
Medical insurance	Fully self-funded					
	Partially medical insurance	0.33	0.32	1.39 (0.74, 2.61)	1.035	0.301
	Fully medical insurance	0.42	0.49	1.52 (0.58, 3.97)	0.860	0.390
Procedure type	Posterolateral fusion					
	Posterior lumber interbody fusion	0.69	0.36	1.99 (0.99, 4.01)	1.920	0.055
	Transforaminal lumbar interbody fusion	0.52	0.34	1.68 (0.87, 3.24)	1.543	0.123
	Anterior lumbar interbody fusion	−0.26	0.33	0.77 (0.40, 1.48)	0.771	0.441
Decompression	Yes					
	No	−0.09	0.27	0.91(0.53, 1.56)	0.339	0.734

### 3.3 Multivariate logistic regression analysis of potential factors associated with early postoperative kinesiophobia

The results of the univariate analysis in Table 2 were used to perform multivariate logistic regression, the results of which are shown in Table 3. Eight associated factors were selected by the model based on stepwise regression: anxiety, depression, self-efficacy (GSES), social support (SSRS), age, sex, VAS, and employment status. Only seven associated factors exhibited significant statistical differences. Further, we manually eliminated the status of employment as it had no significance. The results are shown in Table 4.

**Table 3:** Multivariate logistic regression analysis of the potential predictive factors associated with kinesiophobia in patients with LDDs.

Variables	Variable levels	Beta	SE of Beta	OR	Statistic	*P*
Intercept		−3.43	2.85		1.204	0.229
HADS_Anxiety		0.23	0.08	1.26 (1.09, 1.46)	3.086	0.002
HADS_Depression		0.17	0.06	1.18 (1.04, 1.34)	2.654	0.008
GSES		−0.24	0.06	0.79 (0.70, 0.88)	4.154	<0.001
SSRS		−0.19	0.04	0.82 (0.77, 0.88)	5.497	<0.001
Age		0.16	0.04	1.18 (1.08, 1.28)	3.817	<0.001
VAS		0.52	0.19	1.68 (1.17, 2.41)	2.797	0.005
Sex	Female					
	Male	1.63	0.68	5.12 (1.36, 19.37)	2.409	0.016
Work status	Employed					
	Unemployed	−1.58	0.83	0.21 (0.04, 1.04)	1.908	0.056
	Retirement	−0.24	1.22	0.79 (0.07, 8.60)	0.194	0.846

**Table 4:** Multivariate logistic regression analysis of the potential predictive factors associated with kinesiophobia in patients with LDDs.

Variables	Variable levels	Beta	SE of Beta	OR	Statistic	*P*
Intercept		−3.21	2.22		1.446	0.148
HADS_Anxiety		0.22	0.07	1.24 (1.08, 1.43)	3.096	0.002
HADS_Depression		0.18	0.06	1.19 (1.06, 1.35)	2.898	0.004
GSES		−0.24	0.06	0.78 (0.70, 0.88)	4.328	<0.001
SSRS		−0.18	0.03	0.84 (0.79, 0.89)	5.484	<0.001
Age		0.14	0.03	1.15 (1.09, 1.22)	4.999	<0.001
VAS		0.53	0.18	1.70 (1.19, 2.42)	2.892	0.004
Sex	Female					
	Male	1.42	0.63	4.16 (1.21, 14.27)	2.264	0.024

The values of the Akaike information criterion (AIC) for the model of multivariate logistic regression with and without the employment status were 101.41 and 102.25, respectively. The analysis of variance of the two models showed no statistically significant difference between them (*F* = −4.83, *P* = 0.09). According to the AIC values, introducing the subjects’ employment status may further optimize the model. Thus, we can conclude that anxiety, depression, GSES, SSRS, age, sex, and VAS were the independent factors influencing kinesiophobia.

In particular, the subjects' age [OR = 1.15, 95% Cl = (1.09, 1.22), *P* < 0.001], anxiety [OR = 1.24, 95% Cl = (1.08, 1.43), *P* < 0.05], depression [OR = 1.19, 95% Cl = (1.06, 1.35), *P* < 0.05], and pain [OR = 1.70, 95%Cl = (1.19, 2.42), *P* < 0.05] were positively correlated with kinesiophobia. Social support for the subjects [OR = 0.84, 95% Cl = (0.79, 0.89), *P* < 0.001] and their self-efficacy [OR = 0.78, 95% Cl = (0.70, 0.88), *P* < 0.001] were negatively associated with postoperative kinesiophobia. Males [OR = 4.16, 95% Cl = (1.21, 14.27), *P* < 0.05] were more likely to develop kinesiophobia than females.

The *DAAG* package was used to analyze issues of collinearity among the above variables, and the results are shown in Table 5. The variance inflation factor (VIF) was used to detect collinearity. The VIF values of all variables were smaller than 10, indicating that there was no issue of collinearity involving the variables.

**Table 5:** Collinearity analysis of variables in the multivariate logistic regression model.

Variables	VIF
HADS_Anxiety	1.09
HADS_Depression	1.19
GSES	1.18
SSRS	1.41
Age	1.34
VAS	1.11
Sex	1.02

VIF, variance inflation factor.

### 3.4 Model to identify early postoperative kinesiophobia in patients with LDDs

Based on the results of multivariate logistic regression given in Table 3, the authors chose anxiety, depression, self-efficacy, social support, age, sex, VAS, and employment status as the associated factors to establish a model to identify early postoperative kinesiophobia in patients with LDDs. It can be expressed as follows: *p* = (exp(*x*))/(1 + exp(*x*)), where *x* = −3.21 + (0.22 × HADS_Anxiety) + (0.18 × HADS_Depression) + (−0.24 ×  GSES) + (−0.18 × SSRS) + (0.14 × Age) + (0.53 × VAS) + (1.42 × Sex). The value −3.21 in this formula represents the intercept. The reference level for sex is “Female”. The units/scaling for the continuous predictors in this expression are the age per year, VAS per point, and scale totals per point.

**Figure 2:**
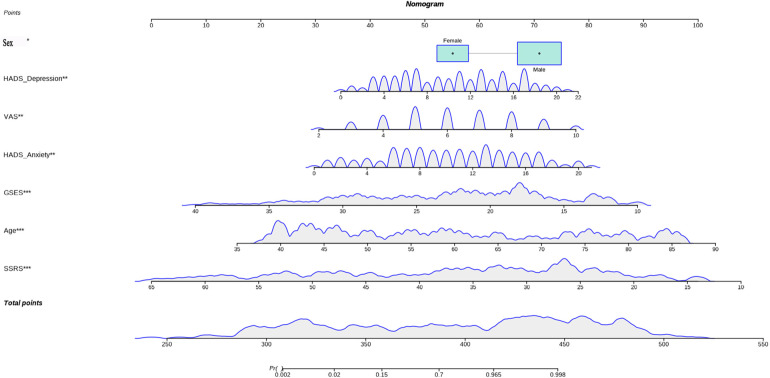
Nomogram for predicting kinesiophobia in patients with lumbar degenerative diseases.

The model was visualized as the nomogram shown in Figure 2. To improve its applicability and convenience of use, we provided a network calculator at https://fangzhumei.shinyapps.io/DynNomapp/. It can be used to easily calculate the probability of early postoperative kinesiophobia.

### 3.5 Evaluation of proposed model

**Figure 3:**
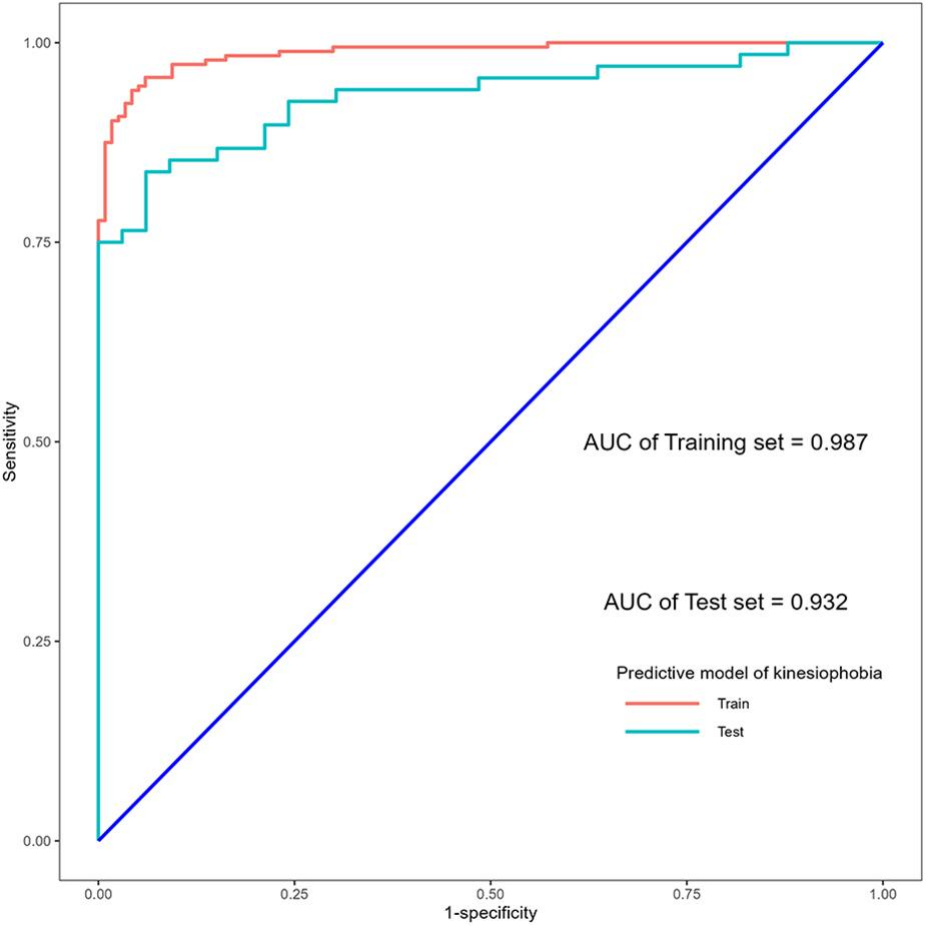
ROC curves of the predictive model for kinesiophobia in patients with lumbar degenerative diseases.

The confusion matrix of the proposed stepwise regression-based model on the training and test sets is shown in Table 6. It was used to determine the precision of the model on both sets, and the results are shown in Table 7. They showed that the stepwise regression-based model had a high precision on both the training and test sets, with recall values of 0.957 (0.917, 0.978) and 0.838 (0.733, 0.907), respectively. They suggested that the model correctly identified early postoperative kinesiophobia in patients with LDDs, with values of precision of 95.7% and 83.8%. The area under the curve, accuracy, sensitivity, and specificity of the model on the training and test sets were 0.931 (0.890, 0.972) and 0.874 (0.781, 0.966), 0.937 (0.904, 0.959) and 0.861 (0.781, 0.916), 0.957 (0.917, 0.978) and 0.838 (0.733, 0.907), and0.906 (0.839, 0.947) and 0.909 (0.764, 0.969), respectively. The other indicators of evaluation are listed in Table 7. The receiver operating characteristic (ROC) curves of the model for the training and test sets are also plotted in Figure 3. The values of these indicators suggested that the proposed model was stable and reliable.

**Table 6:** Confusion matrix of the predictive model for kinesiophobia for the training and test sets.

Training set	Predictive kinesiophobia			Test set	Predictive kinesiophobia		
Actual kinesiophobia		No	Yes	Actual kinesiophobia		No	Yes
	No	106	11		No	30	3
	Yes	8	176		Yes	11	57

**Table 7:** Assessment of the precision of the predictive model for kinesiophobia in patients with LDDs.

Indicators	Training set	Test set
AUC	0.931 (0.890,0.972)	0.874 (0.781,0.966)
Sensitivity	0.957 (0.917, 0.978)	0.838 (0.733, 0.907)
Specificity	0.906 (0.839, 0.947)	0.909 (0.764, 0.969)
Accuracy	0.937 (0.904, 0.959)	0.861 (0.781, 0.916)
Negative predictive value	0.930 (0.868, 0.964)	0.732 (0.581, 0.843)
Positive predictive value	0.941 (0.898, 0.967)	0.950 (0.863, 0.983)
Recall	0.957 (0.917, 0.978)	0.838 (0.733, 0.907)
F-Measure (F1 value)	0.949	0.891
Kappa	0.867 (0.809, 0.925)	0.703 (0.562, 0.845)
Youden index	0.863	0.747

**Figure 4:**
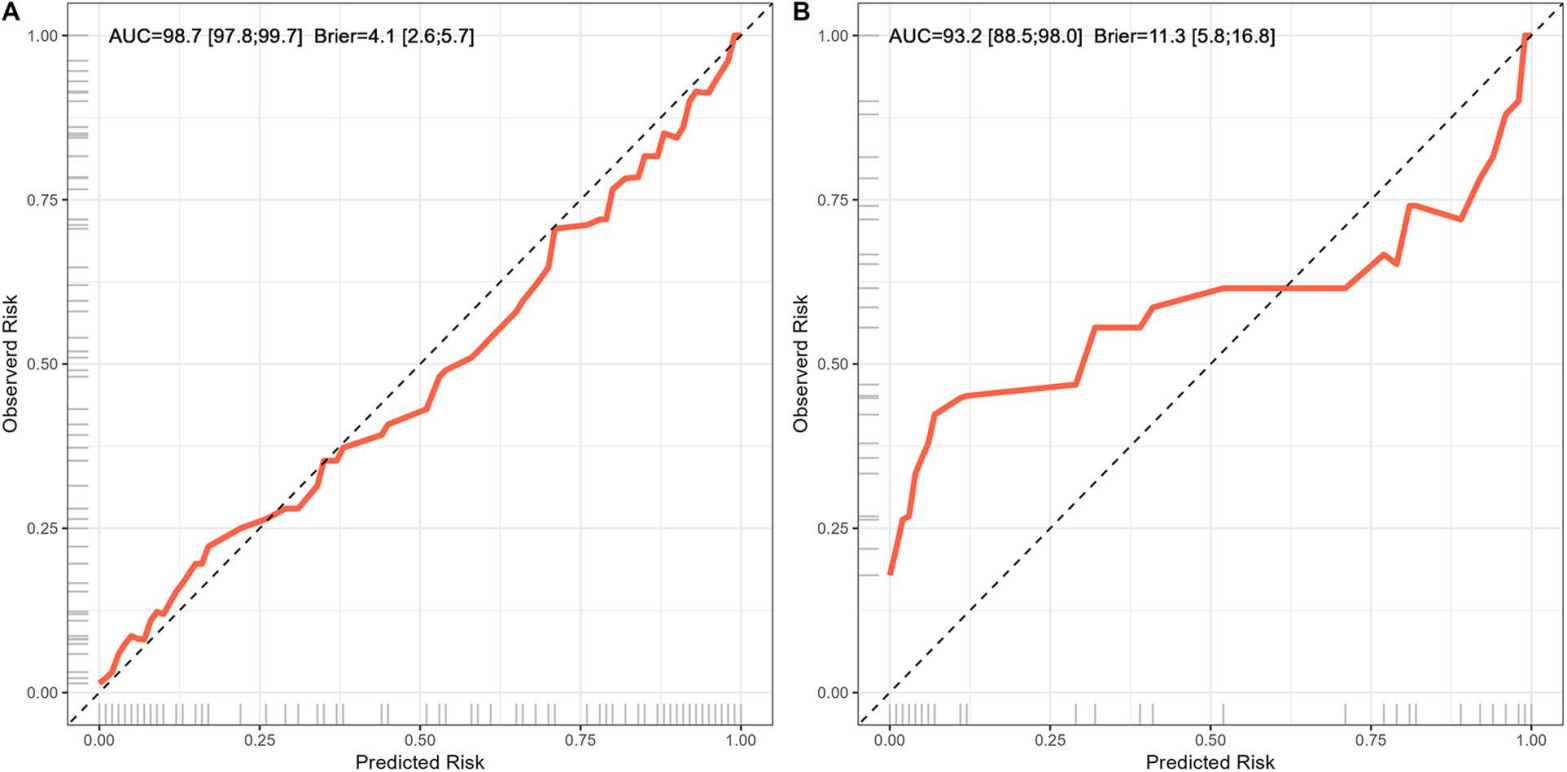
Curve of calibration of the predictive model for kinesiophobia in patients with lumbar degenerative diseases. (A) Curve of calibration in training set. (B) Curve of calibration in test set.

Furthermore, the curves of calibration and Hosmer–Lemeshow test were used to assess the capability of the model for calibration, while the bootstrap resampling method (*B* = 1,000) was used to calculate the values of its parameters. The curves of calibration are shown in Figure 4. The values of the Hosmer-Lemeshow test on the training and test sets were *χ*^2^ = 8.32, *df* = 13, and *P* = 0.822, and *χ*^2^ = 11.63, *df* = 13, and *P* = 0.235. This showed that there was no significant difference between the probability of kinesiophobia in patients with LDDs predicted by the proposed model, and its actual probability.

**Figure 5:**
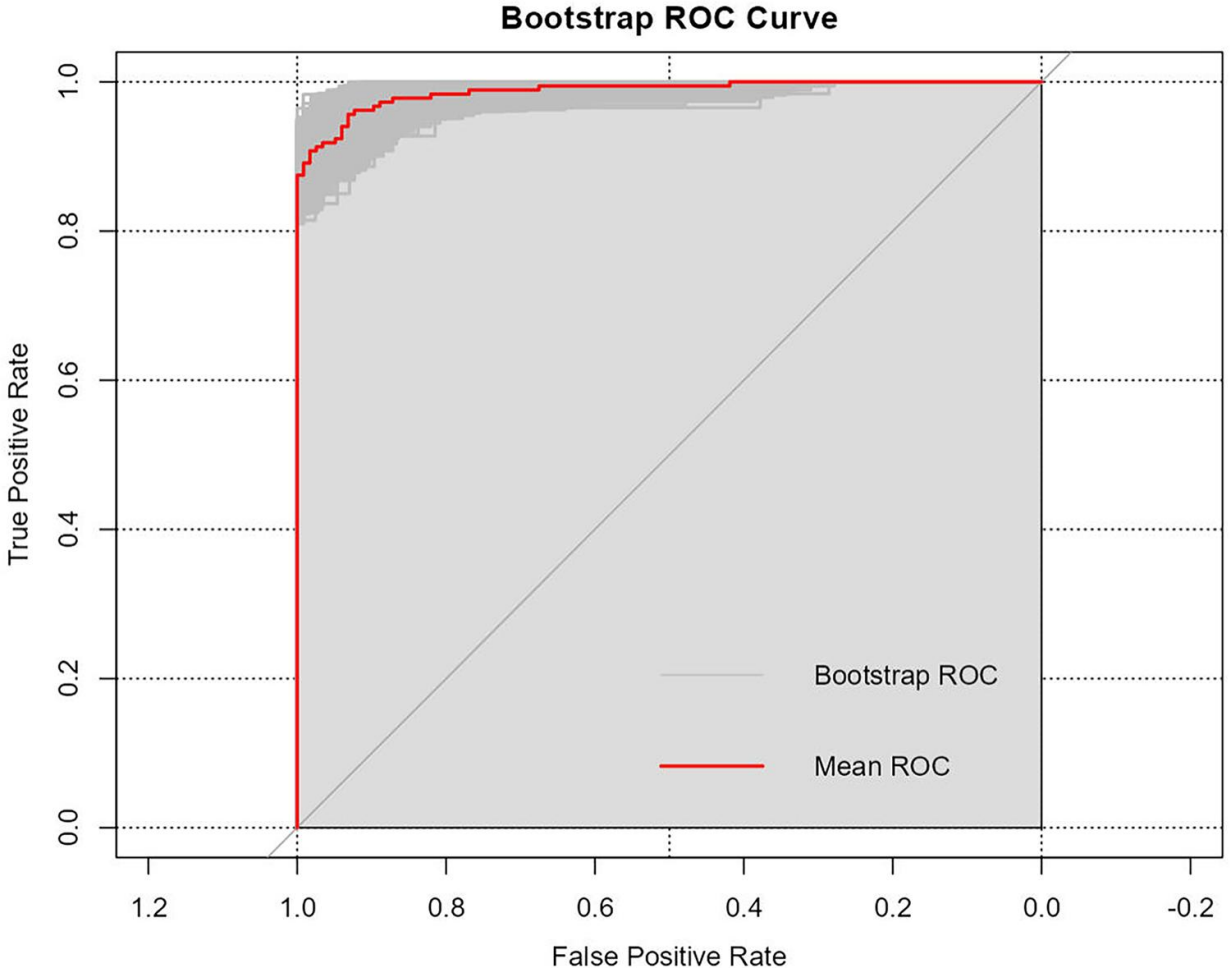
ROC curve of bootstrap re-sampling.

We used bootstrap re-sampling (1,000 replications) to internally validate the model. Its performance-related metrics had the following values: accuracy = 0.924, kappa = 0.840, ROC = 0.981, sensitivity = 0.899, specificity = 0.941, optimism-corrected AUC = 0.988 (0.978, 0.997). The ROC curve of the bootstrap re-sampling is shown in Figure 5.

### 3.6 Clinical application of proposed model

**Figure 6:**
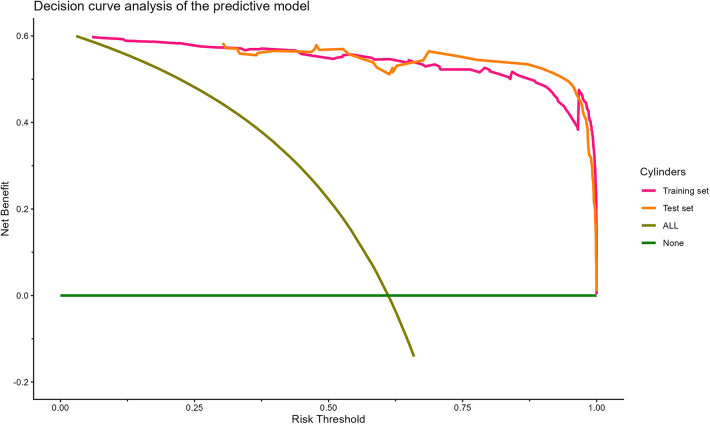
Decision curve analysis (DCA) of the predictive model for kinesiophobia in patients with lumbar degenerative diseases. The *x*-axis represents the threshold probability of patients diagnosed with kinesiophobia, while the *y*-axis represents the net benefit rate. The solid green line represents the extreme case of assuming that neither the training sample nor the test sample reveals kinesiophobia, that is, the net benefit rate is zero. The solid brown line represents the other extreme case, whereby all samples in the training or test set are diagnosed as kinesiophobia, and the net benefit rate is the maximum. The orange and purple lines denote the actual benefits for different cohorts of patients.

**Figure 7:**
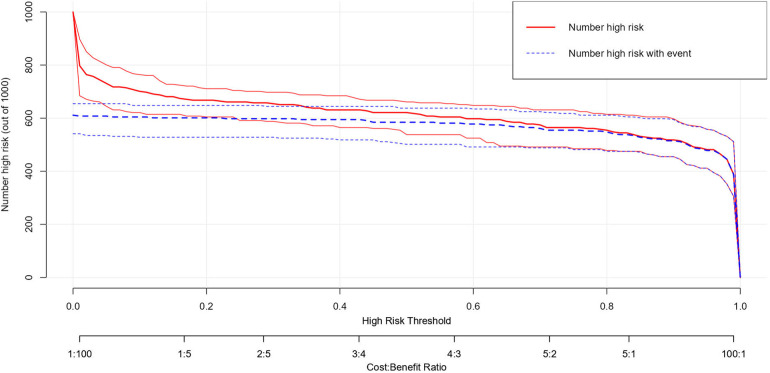
Clinical impact curve (CIC) of the predictive model for locoregional recurrence.

The DCA was used to assess the clinical application of the predictive model (Figure 6). The curve of DCA showed that it provided a greater net benefit and a wider range of threshold probabilities to screen for early postoperative kinesiophobia on the training set compared with the test set. The DCA clearly demonstrated that our model offers greater benefits than both “full treatment” (brown line) and “no treatment” (horizontal, solid, green line) across the thresholds of probability of (1.53%–100.00%) on the training set and (2.08%–100.00%) on the test set. This suggests that the model has some scope for use in clinical applications. Furthermore, the clinical impact curve (Figure 7) showed that the model had a high clinical efficacy, and could thus help nurses quickly screen for early postoperative kinesiophobia in patients with LDDs.

## 4 Discussion

This study has proposed an accurate model to identify early postoperative kinesiophobia in patients with LDDs. The work here provides two innovations in research in the field. First, we successfully identified seven important factors influencing early postoperative kinesiophobia in such patients: namely, anxiety, depression, self-efficacy (GSES), social support (SSRS), age, sex, and VAS. The model was then developed based on these indicators, and delivered excellent performance.

### 4.1 Prevalence of kinesiophobia in patients with LDDs

LDDs are very common, and are primarily treated through surgery. However, the pain experienced by patients with LDDs after surgery is likely to cause kinesiophobia during their postoperative rehabilitation. Research has shown that kinesiophobia is an independent factor that directly contributes to prolonged pain, joint hypoplasia, and incapacitation in patients with LDDs after surgery. It thus impacts the outcomes of rehabilitation (13). In this study, the prevalence of kinesiophobia in patients with LDDs was found to be 62.69% (252/402), higher than has been reported in previous studies (13, 25, 26). This may be related to the different countries in which the studies were conducted, the course of the diseases, and the sample size.

### 4.2 Analysis of independent factors influencing kinesiophobia in patients with LDDs

#### 4.2.1 Age

The authors found that age was an independent risk factor that significantly influenced the development of kinesiophobia. The risk of kinesiophobia increased with age, with OR = 1.18% and 95% Cl = (1.08, 1.28). This is consistent with the findings of Roelof et al. (27). Age is an independent risk factor owing to the special physiological and psychological characteristics of the elderly. As life expectancy has increased, the prevalence of LDDs has grown in the elderly population. The stability of joints of elderly patients decreases, as does their capability for repair, such that aging people are likely to experience more complications, longer recovery times, and a higher prevalence of pain after surgery. Moreover, elderly people are more likely to be fatigued due to the decline in the physical functions and protective mechanisms of their neuromuscular system. They are hindered by their mental state and cognitive function, and are apt to lack confidence in their physical condition. Thus, they are more likely to reduce the intensity of their exercise to avoid a recurrence of their injury. Medical staff should therefore pay more attention to the mental state of elderly patients and their rehabilitation training, and should adopt targeted measures of psychological care according to their specific situation, improve their understanding and ability to cope with kinesiophobia, and encourage them to carry out postoperative rehabilitation with a positive and proactive attitude.

#### 4.2.2 Sex

A total of 64.43% of the participants of this study were male. It was also established that males were more likely to develop kinesiophobia than females, with OR = 5.12(1.36, 19.37). This was similar to the results reported by Misterska et al. (28). Some studies have shown that the prevalence of kinesiophobia in male patients is significantly higher than that in females, such that sex may be a predictor of the disease (27). Male patients who make a living from physical labor and suffer from spinal diseases are usually the breadwinners for their families (29). If they fall ill, their ability to work decreases along with their family income. The cost of treatment in hospital adds to the pressure on family finances, which can in turn easily cause negative emotions, such as anxiety and depression, that can lead to kinesiophobia (30).

#### 4.2.3 Anxiety

The results of this study showed that symptoms of anxiety were positively correlated with kinesiophobia, and constituted an independent risk factor for it in patients with LDDs. This finding was consistent with work by Svensson et al. (13). In addition to pain, preoperative anxiety is often influenced by the following factors: the fear of major surgery, lack of knowledge of surgery, and lack of understanding of such behaviors as perioperative rehabilitation and exercise. In most cases, patients experience stronger physiological and psychological stress reactions before surgery that can easily cause preoperative anxiety (31). In addition, some doctors may overemphasize intraoperative risks and postoperative complications during preoperative conversations, which can further aggravate the patient's anxiety. Some studies have shown that anxiety tends to cause sleep disorders, which in turn cause nociceptive sensitization and increased postoperative pain through the sharing of the pathway for the hypothalamic-pituitary-adrenergic axis. This increases the patient's sensitivity to surgical stress and other discomforts, and reinforces their defense mechanism to induce a greater sense of pain and catastrophic thoughts. This is a considerable psychological burden (31, 32).

#### 4.2.4 Depression

As a psychological manifestation that is highly correlated with anxiety, depression often generates the same clinical consequences. Depressive symptoms were found to be positively correlated with kinesiophobia in this study. It was an independent risk factor that is related to kinesiophobia in patients with LDDs. In general, pain and depression have a common pathway for neurotransmission, and activate the same area of the brain such that this changes the learning and cognitive abilities of the individual, enhances their perception of pain, and aggravates their fear of rehabilitation training (33). The negative psychological states of kinesiophobia, anxiety, and depression interact with one another (31). Patients with LDDs who endured pain for a long time were prone to anxiety and depression, which led to the avoidance and fear of rehabilitation exercises. This in turn resulted in reduced social activities and weakened muscles. The consequent reduced mobility further exacerbated the symptoms of anxiety and depression, to create a vicious cycle.

Therefore, medical staff should pay attention to kinesiophobia due to anxiety and depression among patients, seek to allay such negative psychological emotions, change the patients' negative understanding of pain and rehabilitation activities, and help them break free from the vicious cycle of kinesiophobia. Measures for patients with anxiety and depression should include stress reduction, individualized relaxation training, and music therapy to help control their negative emotions. Moreover, the staff should educate patients on LDDs and kinesiophobia.

#### 4.2.5 Self-efficacy

Self-efficacy refers to an individual's expectations, perceptions, beliefs, or confidence about their own ability to control their behavior and the environment needed to accomplish a specific purpose (34). It reflects the level of confidence of patients in effectively managing their clinical symptoms, and engaging in rehabilitation activities and exercise (35). This study revealed that self-efficacy was a negative moderator that significantly influenced kinesiophobia. Patients with LDDs who had a lower self-efficacy had a higher risk of postoperative kinesiophobia. Self-efficacy can be used to determine the effort and time consumed by an individual when they encounter difficulties or obstacles, and thus influences whether a patient can overcome their fear of movement after surgery. Patients with a higher self-efficacy are more willing to overcome such stimuli as severe pain after surgery, rather than seeing the pain as an injury to the body or as a threat to be avoided, and are more willing to respond proactively. Patients with a higher self-efficacy are therefore more proactive in implementing healthy behaviors (36). Self-efficacy has been found to mediate pain-induced motor fear and avoidance-related behaviors, while patients with a low self-efficacy have twice the risk of disability than those with a high self-efficacy (37). Therefore, it is important to improve the self-efficacy of patients with LDDs during surgical treatment and rehabilitation by distributing brochures on health education to them, providing detailed explanations of diseases, formulating individualized plans for rehabilitation, motivating patients with successful cases, and affirming and encouraging their progress. Strengthening the patients' confidence in defeating the disease can help them overcome their fear of exercise, and can enable them to take the initiative to engage in rehabilitation exercises.

#### 4.2.6 Social support

The results of this study have shown a significant negative correlation between social support and kinesiophobia. Patients with strong social support have a low risk of developing kinesiophobia. Social support refers to the help provided by the patient's family and society at large, and includes material and emotional support as well as information and companionship (38). In most cases, the family members of the subjects of this study not only participated in medical decision-making during their hospitalization, but also shared the task of postoperative rehabilitation management. Most of the subjects in this study were elderly, and family health educators were the main source of social support for patients. Postoperative patients with LDDs had a poor ability for self-care, had undergone significant physical and psychological changes, and were subject to many adverse stimuli, including pain, anxiety, and depression. Thus, they needed care and support from their family members. The more support that a patient perceived from their family members, the stronger was their sense of security, the more positive was their mindset, and the better they could cope with their situation (39). Strong familial support can improve the patient's cognition of pain and activities, relieve adverse emotions in a timely manner, help them establish a strong psychological defense mechanism, reduce the occurrence of emergencies, and help them face stress in a positive manner. Patients often choose not to confide in their family members to avoid worrying them. This leads to a low level of social support for them, and makes them prone to harbor negative emotions and be fearful of secondary injuries caused by physical activity such that they avoid exercise.

#### 4.2.7 Intensity of pain

This study showed that the intensity of pain was an independent risk factor for predicting kinesiophobia. For patients with LDDs, the intensity of pain increased the risk of developing kinesiophobia after surgery by OR = 1.68 (1.17, 2.41). Previous studies have demonstrated a significant positive correlation between the level of pain and kinesiophobia (40). Although surgical treatment could significantly improve lumbar spine function in patients with LDDs, the painful sensation of the surgical wound made patients feel that it was harmful to the body. At the same time, the pain experienced by them during postoperative rehabilitation training could easily cause patients to develop a fearful mentality and become unwilling to undertake further activities. The lack of early functional exercise can lead to the disuse of limb, which affects the recovery of the lumbar spine function, and leads to recurrent pain and an increased need for medical treatment (40). In addition, pain also triggers such adverse emotions as anxiety and depression, which reduce the patients' pain threshold and aggravate symptoms of fear. Therefore, medical staff should closely monitor the patients' complaints of pain before and after surgery, attend to their level of pain, reorient their understanding of pain, and provide medication and treatment under the guidance of doctors. Furthermore, patients should be instructed to use the pain diary method, music therapy, and methods of stress reduction to control their level of pain and, thus, kinesiophobia.

### 4.3 Development and application of the proposed model

This study developed a nomogram for identifying early postoperative kinesiophobia in patients with LDDs who had undergone surgery. To the best of the authors' knowledge, this is the first model of its kind to have been proposed in the literature. The findings identified the independent factors associated with kinesiophobia in the studied population: age, sex, intensity of pain, anxiety, depression, self-efficacy, social support, and employment status. The nomogram constructed based on these associated factors exhibited sound performance following external validation.

The proposed model accurately identified patients at high risk of kinesiophobia, which was useful for clinical prevention and treatment. The nomogram was integrated with the available scales of assessment for kinesiophobia to comprehensively evaluate patients with LDDs. For example, a patient with LDDs who had no symptoms of kinesiophobia scored below 37 points on the TSK. However, this score did not eliminate the possibility of their developing kinesiophobia in the future. To address this gap in assessment, our web-based calculator was used to quantify the patient's probability of developing kinesiophobia. It supplemented existing scales for screening kinesiophobia, and helped physicians and nurses identify beneficial intervention strategies for patients with LDDs who are at risk of kinesiophobia.

### 4.4 Model-guided nursing intervention

The DCA in Figure 6 clearly shows that our model offers greater benefits than both “full treatment” (brown line) and “no treatment” (horizontal, solid, green line) over thresholds of probability of (1.53%–100.00%) on the training set and (2.08%–100.00%) on the test set. Given thresholds with these two ranges, postoperative care should ensure better outcomes of intervention. The optimal nursing interventions for kinesiophobia remain unclear (20). Additional counseling, cognitive and behavioral therapy, and education or tailored mobilization may be options to consider. Our findings here can help determine the optimal timing for intervention in such cases.

### 4.5 Strengths and limitations

The clinical screening model established in this study made the following contributions to the literature: First, no previous study has reported the use of nomograms to identify early postoperative kinesiophobia in patients with LDDs who have undergone surgery. Second, the proposed model exhibited sound performance in terms of discrimination, calibration, and clinical applicability. The network calculator used multivariate logistic regression for ease of clinical application. Our model provided a convenient tool for the early screening of early postoperative kinesiophobia so that interventions may be made as needed. Preventing postoperative kinesiophobia in patients with LDDs could improve their in-patient experience and clinical outcomes. By leveraging the proposed predictive model, nurses could forecast the onset of postoperative kinesiophobia, promptly identify high-risk individuals, and facilitate early diagnosis and the formulation of targeted interventions for kinesiophobia to improve the quality of healthcare provided. Furthermore, the proposed model could help reduce the cost of healthcare and avoid the waste of resources by minimizing unnecessary interventions.

However, there were many limitations in the work presented here. First, we adopted a cross-sectional design, which made it difficult to determine the temporal relationships between variables. Consequently, the findings concerning the factors influencing kinesiophobia may be subject to bias, and future studies should apply prospective designs to address this issue. Because the predictors and outcomes were reported by the patients and collected concurrently in this cross-sectional study, the observed relationships between them may reflect shared methodical variance and bidirectionality. Second, the clinical model contained some psychological factors, the indicators of the outcomes of which could not be directly measured. They needed to be assessed via questionnaires, where this may introduce some subjectivity to the results. Third, our model requires scales containing several items (HADS, GSES, SSRS) as well as the VAS and patient demographics. While it may be feasible to acquire this information in research settings, the burden of daily work in hospitals makes it unviable in practice. Perhaps future research can develop a simpler model with fewer predictive factors based on our model. Fourth, the external validity and clinical interpretation of our model may be limited because we ignored certain influential factors. For example, the number of lumbar levels, distribution of pathologies, protocol for perioperative analgesia, pathway of mobilization or rehabilitation, and major early complications or ICU transfers may also influence the occurrence of kinesiophobia. Finally, due to constraints on funding, this study covered only two hospitals and recruited the participants by using convenience sampling, which may limit the generalizability of the model.

## 5 Conclusion

The proposed model used the age, sex, intensity of pain, anxiety, depression, self-efficacy and social support of patients with LDDs to identify early postoperative kinesiophobia in them. It exhibited sound performance in terms of discrimination, calibration, and clinical applicability, and accurately identified early postoperative kinesiophobia in patients with LDDs. Furthermore, the nomogram model and online web-based calculator were easy to use for nurses, and enabled them to quickly identify early postoperative kinesiophobia in patients with LDDs.

## Data Availability

The original contributions presented in the study are included in the article/Supplementary Material, further inquiries can be directed to the corresponding authors.
